# The multifaceted nature of endogenous cardiac regeneration

**DOI:** 10.3389/fcvm.2023.1138485

**Published:** 2023-03-14

**Authors:** Laura Rolland, Chris Jopling

**Affiliations:** Institute of Functional Genomics, University of Montpellier, CNRS, INSERM, LabEx ICST, Montpellier, France

**Keywords:** heart, regeneration, inflammation, cardiomyocyte, zebrafish, neonatal mouse, hypoxia

## Abstract

Since the first evidence of cardiac regeneration was observed, almost 50 years ago, more studies have highlighted the endogenous regenerative abilities of several models following cardiac injury. In particular, analysis of cardiac regeneration in zebrafish and neonatal mice has uncovered numerous mechanisms involved in the regenerative process. It is now apparent that cardiac regeneration is not simply achieved by inducing cardiomyocytes to proliferate but requires a multifaceted response involving numerous different cell types, signaling pathways and mechanisms which must all work in harmony in order for regeneration to occur. In this review we will endeavor to highlight a variety of processes that have been identifed as being essential for cardiac regeneration.

## Introduction

Myocardial infarction (MI), or heart attack, combined with strokes, accounted for more than 15 million deaths worldwide in 2019 (1). It consists of the interruption of the blood flow in one of the coronary arteries. In most cases, it is the result of atherosclerosis and more specifically the rupture of an atherosclerotic plaque blocking an artery. The first consequence of the rupture is ischemia, a lack of blood supply leading to hypoxia, affecting the region of cardiac tissue normally supplied by the artery. This region is then defined as the infarcted area and is associated with necrosis. The loss of cardiomyocytes due to the ischemic episode is followed by a period of remodeling. This is associated with excessive extracellular matrix (ECM) deposition including collagen, forming a scar in place of the healthy tissue, a compensatory mechanism set up to repair the damaged heart. Overall, it leads to thinning of the ventricular wall and dilation, accompanied by wall stress disruptions and impaired cardiac function (2). Signaling pathways triggered by either neuroendocrine hormones (produced in response to the injury) or disrupted mechanical forces will eventually cause cardiomyocyte hypertrophy (3, 4). This pathological remodeling and the underlying mechanisms, cannot at present be overcome and eventually will lead to heart failure, associated with a high risk of mortality (5). Certain organisms, however, avoid such adverse effects after an injury as they are able to completely regenerate their heart.

**Figure 1:**
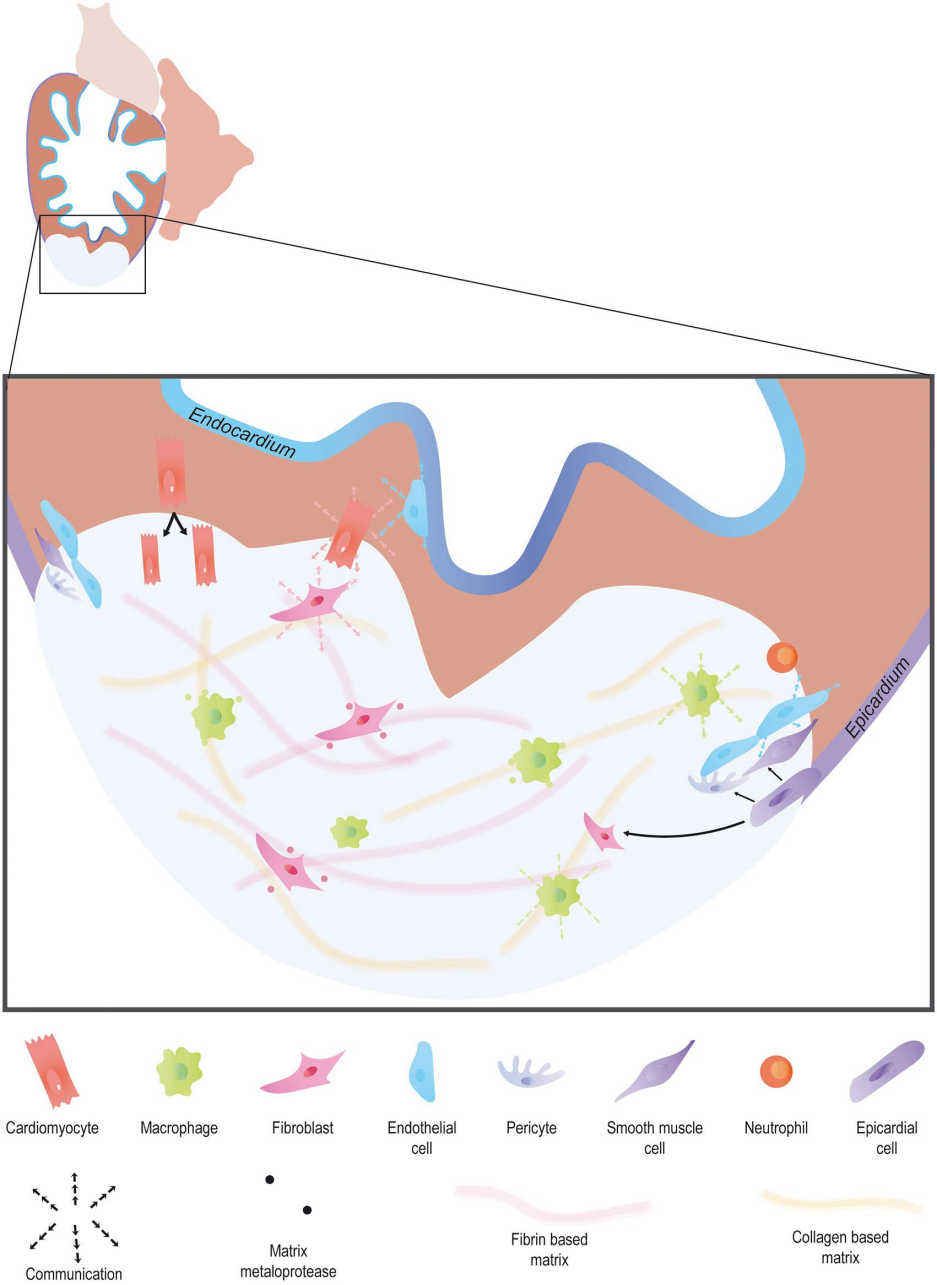
Endogenous heart regeneration. Following an injury, the majority of cardiac interstitial cells are involved in the process of regeneration. Immune cells invade the wound to clear debris and secrete cytokines to promote the fast resolution of the pro-inflammatory phase. Pro-angiogenic factors are secreted by a large variety of cardiac cell types to promote angiogenesis. Activated epicardial cells undergo EMT, forming epicardium-derived cells including fibroblasts, and vascular cells. In turn, fibroblast activation allows matrix remodeling, first *via* the secretion of a transient collagen-based matrix which is then degraded leaving room for a fibrin-based matrix. Finally, cardiomyocytes proliferate, forming new and functional myocardial tissue.

Cardiac regeneration has been studied for decades now. The first evidence dates back to 1974, with the discovery that adult newts were capable of regenerating their heart in response to ventricle resection (6). It took almost 30 years after that to show similar results in adult zebrafish in response to the same injury (7). More recently neonatal mice have been shown to possess cardiac regenerative capacities after an injury (8). Since then, many studies have focused on cardiac regeneration in both mammals and non-mammalian species because of the therapeutic potential of the mechanisms involved in this process. It is now apparent that cardiac regeneration is not simply achieved by inducing cardiomyocytes to proliferate but requires a multifaceted response involving different cell types, signaling pathways and mechanisms which must all work in harmony in order for regeneration to occur (Figure 1). In this review, we will endeavor to highlight the numerous processes that have been identifed as being essential for cardiac regeneration.

## Models

### Zebrafish

In 2002, the first evidence of cardiac regeneration in zebrafish was provided. This demonstrated that the adult zebrafish heart could almost fully regenerate 30 days after resection of the ventricle apex or amputation (dpa) (7). Subsequently, the mechanism by which zebrafish achieve this remarkable feat was identified. Rather than employing stem/progenitor cells it appears that the zebrafish heart regenerates by the dedifferentiation, proliferation and redifferentiation of pre-existing cardiomyocytes (9).

Since then, other approaches have been used to study heart regeneration in zebrafish beside apical resection such as cryoinjury (10). This involves utilizing a cryoprobe to cause an injury that results in the infarction of a region of the ventricle. A major difference between the amputation and cryoinjury models is that a large apoptotic response occurs after cryoinjury. However, this technique also requires a longer recovery period than apical resection taking upto 60 days post injury (dpi) to see complete regeneration.

Although both techniques represent good injury models to study cardiac regeneration, they require open surgery and therefore, their invasiveness can be seen as a limition. To avoid surgery, genetic ablation can also be performed. This can be achieved using transgenic zebrafish which express Dyphteria toxin A chain (DTA), a cytotoxic agent, in cardiomyocytes following tamoxifen (4-HT) treatment (11). Cardiomyocyte proliferation and regeneration can be observed at 30 days post treatment using this method. However, the non-localized nature of the genetic ablation as well as the cell-type specificity make this technique less representative of MI and is thus less commonly used.

Genetic ablation can also be achieved using the Nitroreductase (NTR)/Metronidazole (Mtz) system, first described in the zebrafish larvae. Similarly to DTA, it allows the ablation of large numbers of cells due to the expression of the *NTR* under a cardiomyocyte specific promoter (12). Once treated with Mtz, NTR converts Mtz into a cytotoxic product, leading to the death of *NTR* expressing cells. This technique was recently utilized to investigate the presence and role of tissue regeneration enhancer elements (TREEs) present in the cardiac *leptin b* enhancer (cLEN) (13). Genetic ablation of cardiomyocytes in either larvae, juvenile or adult zebrafish triggered the activation of cLEN. Furthermore, similar results were observed after cardiac amputation which restricted the activation of cLEN TREEs to the wound area (13). This not only confirmed the validity of genetic ablation to trigger regeneration at all stages of life but also highlighted the impact of regionalized transcriptional activity after an injury, an important feature that cannot be studied using genetic ablation. Interstingly, cLEN activation was later shown to be dependant on mechanical forces as an inhibition of hemodynamic forces [using a *troponin t 2* (*tnnt2*) morpholino (MO) injection], or pharmacological inhibition using 2,3-Butanedione monoxime (BDM) in larvae leads to an impaired activation of cLEN after genetic ablation (14). This raises several interesting questions, regarding the conservation of these regulatory mechanisms in adults, and the potential impact of mechanical forces on their activation in response to an injury.

More recently a similar approach to the NTR system has also been developed which relies on chemoptogenetic abalation of cardiomyocytes (15). This technique relies on the ability of a fluorogen-activating protein (FAP) to bind fluorgenic dyes such as malachite green-ester fluorogen and subseqeuntly produce cytotoxic reactive oxygen species upon illumination with 660 nm light. By driving expression of FAP using a cardiomyocyte specific promoter in zebrafish it is possible to induce wide spread cardiomyocyte abalation which triggers the remaining cardiomyocytes to proliferate and regenerate the damaged myocardium. Furthermore, this does not appear to involve the activation of either the epicardium or endocardium which normaly occurs following apical resection or cryoinjury. Although this system may be useful to focus on the mechanisms which regulate cardiomyocyte proliferation it does not trigger many of the processes which are normally associated with MI in adult mammals. In particular the injury is not localized and does not lead to scar formation and, importantly, scar resorption, both of which will be required *en route* to regenerating an adult mamamlian heart.

All of these techniques/models offer certain advantages and disadvantages which should be taken into account depending on the specific question being asked. For example cryoinjury results in widespread cardiomyocyte necrosis and scarring but takes a significantly longer time to regenerate than the apical resection model. So if the objective is to understand more about the injury/wound healing process then perhaps the cryoinjury model would be more appropriate whereas if the goal is to rapidly identify factors required for succesful regeneration then apical resection would offer a more streamlined approach.

**Figure 2:**
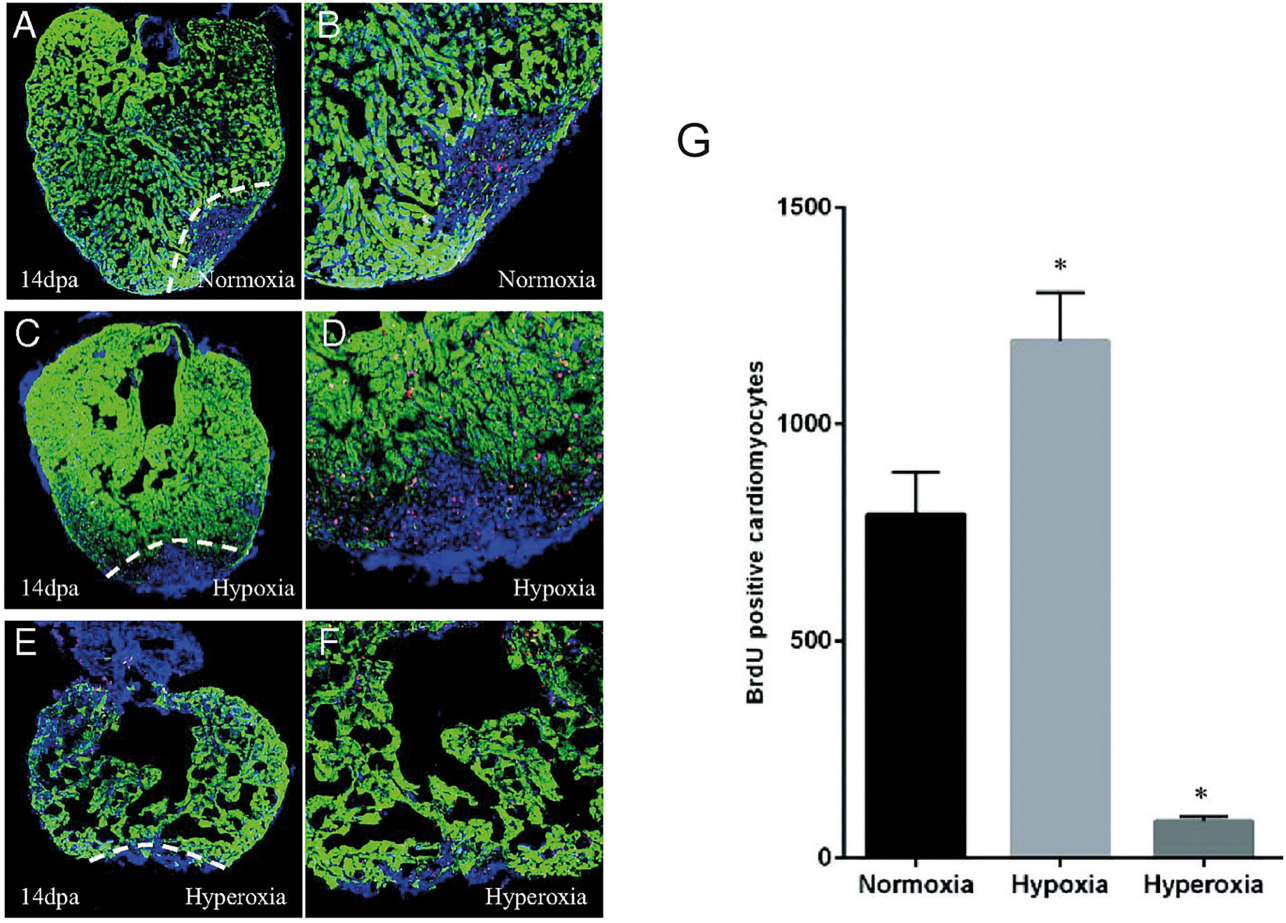
Hypoxia stimulates cardiomyocyte proliferation after injury in zebrafish. A-F. 14 dpa immunofluorescent staining under normoxic conditions **(A,B)**, hypoxia **(C,D)** or hyperoxia **(E,F)** and showing α-sarcomeric actin (green), BrdU (5-bromo-2′-deoxyuridine) (red) and DAPI (blue). **(G)** The number of BrdU-positive proliferating cardiomyocytes increased in hypoxic conditions while it decreased in normoxic conditions. Adapted from Jopling et al. (16).

Another feature associated with cardiac inury which can also play a potential role in regulating cardiac regeneration is hypoxia (16, 17). After cardiac resection in zebrafish there is a strong localized hypoxia induction in the adjacent myocardium. This localized response can be enhanced following the induction of systemic/chronic hypoxia by deleting red blood cells using phenylhydrazine treatment (16). Under hypoxic conditions, cardiomyocyte proliferation is significantly increased whereas under hyperoxic conditions, cardiomyocyte proliferation is reduced leading to impaired heart regeneration (Figure 2). This stresses the importance of hypoxia in the regenerative process and should be taken into account in future regenerative strategies especially as current therapies aim to reduce hypoxia.

Other models, less frequently used, also provide useful insights into the regenerative process such as explant culture, i.e.*, ex vivo* culture of cardiac slices after injury to study the dynamic mechanisms underlying cardiomyocyte division (18). Finally, it is also possible to trigger cardiac regeneration in zebrafish larvae using laser-induced injury (19).

### Mammalian models: A matter of timing

Several mammalian species have been identified as able to regenerate their hearts after an injury in the early stages of life. In particular neonatal mice have been demonstrated to have pronounced albeit short lived cardiac regenerative abilites. Unfortunately this ability to regenerate is lost within the first week of birth. Following cardiac resection of either postnatal day 1 (P1) or P7 mice, complete cardiac regeneration and recovery of cardiac functions at 21dpa occurs in the P1 resected neonates but not in those amputated at P7. This demonstrates that mammals have regenerative abilities during a brief window after birth. Furthermore, cardiac regeneration in neonatal mice is associated with the proliferation of pre-existing cardiomyocytes which occurs after dedifferentiation and sarcomere disassembly similar to what has been described in zebrafish (8). Since then, numerous studies have attempted to extend the regenerative capacity of cardiomyocytes after MI past the P7 regenerative window.

African spiny mice (*Acomys*) display a remarkable ability to regenerate complex tissues such as skin and kidney (20). Recent studies have also determined that *Acomys* is resistant to the damage associated with MI. Indeed *Acomys* display reduced mortality and improved cardiac function following MI when compared to their B6 cousins (20–22). This ability appears to be associated with an enhanced angiogenic response following injury resulting in reduced scarring (20). Furthermore, a significant proportion of *Acomys* cardiomyocytes display a more immature phenotype. In particular around a quarter are mononucleated, a feature that has a significant impact on the ability of cardiomyocytes to proliferate (20). However, whether this ability to resist MI induced damage is due to actual cardiac regeneration remains unclear at present.

Cardiac regeneration has also been observed in large mammals. Neonatal pigs are not only capable of heart regeneration after apical resection performed at P1, but also maintain this ability when MI is induced by left anterior descending (LAD) artery ligation 27 days later, on the same previously amputated animals. These results suggest that preconditioning could extend the regenerative window after birth and it will be interesting to understand the molecular mechanisms associated with this phenomenon (23). But despite the advantages of using large mammalian models as a proxy for humans in terms of physiological parameters, these experiments are costly and time-consuming.

Among the techniques used to induce MI and study heart regeneration, LAD ligation is one of the most commonly used [although it should be noted that in comparison to humans, mouse hearts do not possess a true LAD (24)]. Using this technique on P4 mice, it was shown that although cardiomyocyte proliferation increased 21 days post MI, cardiac hypertrophy was also observed 60 days after injury. This emphasizes how narrow the regenerative window is to achieve complete regeneration in mammals (25).

This approach can also be used to study ischemia reperfusion, by releasing the ligation a few minutes after causing it (26). This technique, more subtle than the permanent ligation is closer to the most prevalent form of MI currently encountered in patients. Indeed, medical progress and rapid patient care shorten the duration of ischemia, thus reducing the extent of the injury. This duration directly correlates with the severity of the infarction: a shorter duration causes subendocardial infarction, affecting only the innermost layers of the myocardium and is often associated with an absence of ST segment elevation in ECG recordings (non STEMI), whereas a longer duration causes total or transmural infarction, associated with an elevation of the ST segment (STEMI) (27).

**Figure 3:**
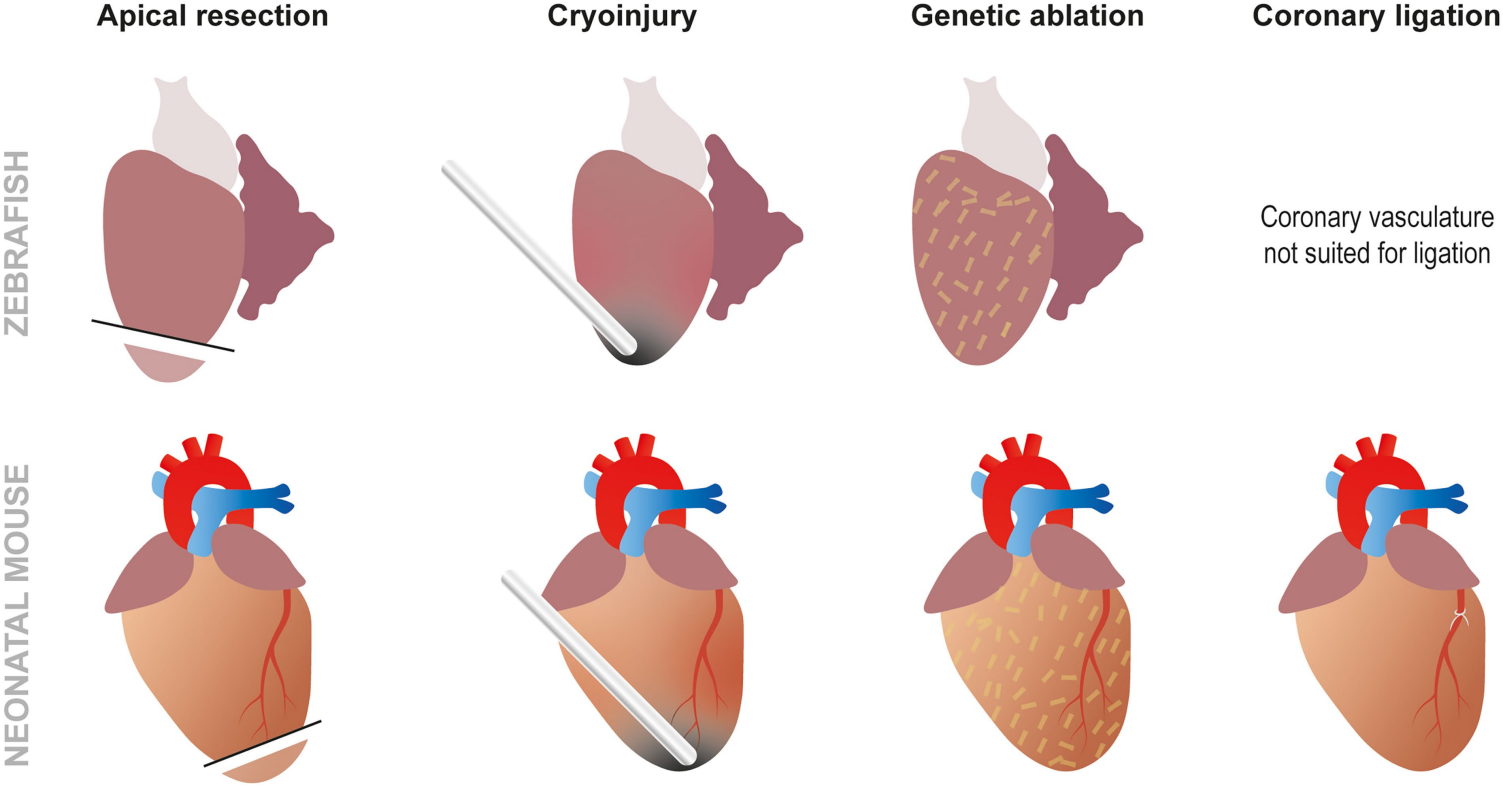
Approaches to study cardiac regeneration. While the most frequently used techniques to study zebrafish heart regeneration are apical resection and cryoinjury, it is also possible to induce genetic ablation using the NTR/Mtz or DTA systems. In neonatal mouse models, the same techniques can also be used but, unlike zebrafish, coronary ligation can also be performed to induced MI.

These methods are especially useful for developing MI in animal models presenting easily discernible coronary vasculature, but the smaller the organism is, the more difficult this gets. That is why other techniques similar to the ones used in zebrafish such as ventricle resection are often used, particularly in small animals like rodent fetuses or neonates (8). Although less commonly employed, the absence of distinct coronary vasculature can also be overcome by using cryoinjury at the apex of the ventricle leading to a similar amount of necrosis and fibrotic scar (28). The regenerative response obtained using this method, however, seems to be controversial as a number of studies show a lack of regeneration when performing cryoinjury as opposed to resection in neonatal mice (29). Finally, genetic ablation can be performed without any form of surgery using the NTR/Mtz system or cell type specific DTA expression. Although these techniques are not frequently used in mammals, specific DTA ablation of cardiomyocytes has been utilized to demonstrate that fetal mice can effectively regenerate cardiomyocytes (30, 31). All these methods, combined with genetic or chemical screening, provide different insights to better understand cardiac regeneration (Figure 3). For example, a recent study has combined the use of several of these approaches to study the role of low-density lipoprotein receptor-related protein 6 (LRP6) involved in the canonical Wnt pathway in heart regeneration. This paper demonstrated that LRP6 was involved in cell cycle inhibition. Subsequently, it appears that inhibiting LRP6 can induce cardiomyocyte proliferation after MI in adult mice. Furthermore, 10% of the newly regenerated cardiomyocytes presented a development-like profile (diploid and mainly mononucleated). In conjunction the animals displayed improved cardiac function, less fibrosis, and no cardiomyocyte hypertrophy. A promising feature of this study for therapeutic applications is the fact that similar results were obtained by either conditional knock out (cKO) or by virally induced knock down (KD). Moreover, the therapeutic effects of LRP6 inhibition can be established post MI making this a viable future therpeutic strategy (32).

Because mammalian neonates lose the ability to regenerate their heart after 1 week the focus is now aimed at understanding what triggers this transition from regenerative to non-regenerative. Transcriptomic analysis has determined that there is an increased expression of *Hoxb13* and *Meis1* transcription factors in P7 mice compared to P1. Indeed, *Hoxb13* KO mice exhibit small proliferative cardiomyocytes. Double KO for both *Hoxb13* and *Meis1* mice present enlarged hearts associated with a high number of small proliferating cardiomyocytes in the absence of injury. After MI, the double KO animals show a signficantly improved recovery suggesting that the HOXB13/MEIS1 complex plays a significant role in arresting cardiomyocyte proliferation in mammals. It also appears that CALCINEURIN [a protein known to be involved in both physiological and pathological hypertrophy (3)] can modulate HOXB13 activity by regulating its phosphorylation status (33).

## Mechanisms underlying heart regeneration

Using these different model systems, several molecular mechanisms associated with heart regeneration have been identified. Initially, the main focus has been directed towards the cardiomyocyte population as this seems to be the main driver of the regenerative process. However, it is now apparent that much more is required than simple cardiomyocyte proliferation and that the cardiac interstitial cells also play a crucial role in providing the optimal environment for cardiomyocytes to act. Although some discrepancies exist between zebrafish and neonatal mice, a common framework appears to be conserved between these species, highlighting the translational potential of zebrafish as a model of cardiac regeneration.

### Cardiomyocytes: The stars of the show

#### Cell cycle reentry, ploidy, and nucleation

**Figure 4:**
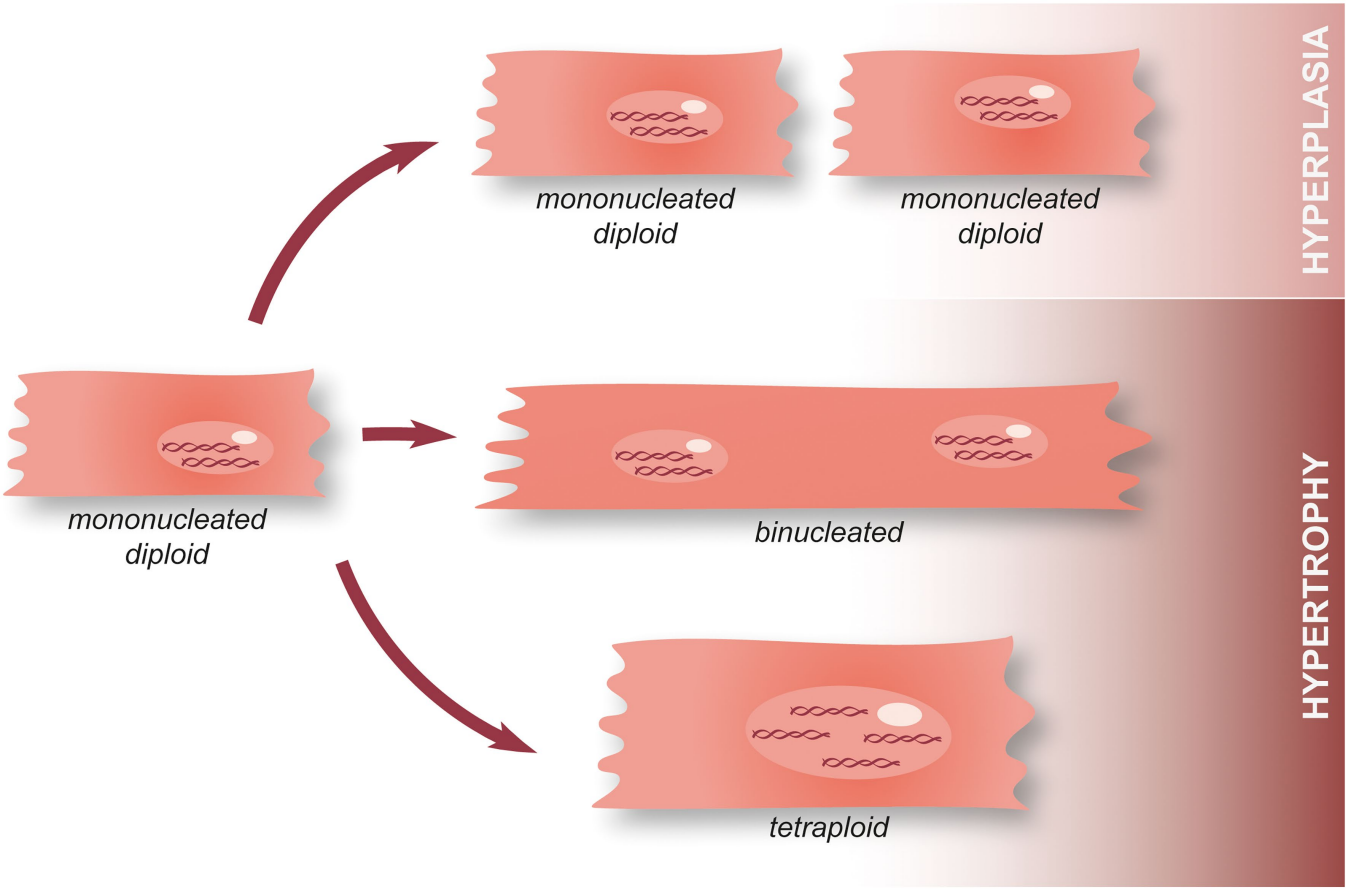
Hypertrophy and hyperplasia in cardiomyocytes. During the first postnatal week, mammalian cardiomyocytes switch from hyperplasia to physiological hypertrophic growth. Following this, a minority of mononucleated diploid cardiomyocytes remain present in the adult heart and can either undergo cell division associated with the annual turnover (hyperplasia), perform acytokinetic mitosis leading to the formation of a binucleated cardiomyocyte, or endoreplication making tetraploid cardiomyocyte. Once they become tetraploid or plurinucleated, cardiomyocytes lose their ability to perform mitosis.

At the cellular level, cardiomyocyte hyperplasia (aka proliferation) and hypertrophy can be seen as two antagonistic processes. The former allows hyperplastic growth, i.e., an increase in the number of cardiomyocytes (cell division and thus, proliferation) while the latter is associated with hypertrophic growth, that is to say an increase in cell size. In this context, it has been shown that cardiomyocyte nucleation and ploidy (the number of complete sets of chromosomes, *n*) is critical (34). Indeed, mononucleated diploid cardiomyocytes have a lot more options regarding their future than tetraploid or binucleated cardiomyocytes (Figure 4). They can either undergo cell division (to produce two mononucleated diploid cardiomyocytes, i.e., hyperplasia), mitosis without cytoplasmic division (acytokinetic division forming a cell containing two diploid nuclei), or endoreplication (leading to the replication of the DNA with no division of the nucleus and creating a tetraploid mononucleated cardiomyocyte) (34). The cells obtained through the last two options are subsequently incapable of entering mitosis and can only undergo endoreplication (Figure 4). Several hypotheses have been formulated to explain this phenomenon for example, the need of an intact centrosome to perform cell division which is not found in binucleated cells, as well as chromosome telomere shortening (causing a failure to transition through cell cycle checkpoints) (34). It is interesting to note that while adult mice hearts are primarily populated with binucleate cardiomyocytes, in the human adult heart, polyploid cardiomyocytes are predominent and binuclear cells only represent 25% (34). This suggests that adult humans may actually already possess an enhanced ability to regenerate their hearts when compared to adult mice. However, it should also be noted that there is still controversy surrounding the idea that mononuclear cardiomyocytes could possess an enhanced regenerative capacity compared to their binucleate counterparts. Previous research has demonstrated that KO of *Tnni3k* (troponin I-interacting kinase) results in an increase in mononuclear diploid cardiomyocytes and subsequent cardiomyocyte proliferation after injury (35). However, in a separate study, an increase in mononuclear diploid cardiomyocytes by KO of *E2F Transcription Factor 7/8* did not result in any noticeable cardiac regeneration after MI.

Although mononucleated diploid cardiomyocytes remain present in the human adult heart, they represent a small fraction of the population in contrast to the zebrafish heart and the developing mammalian heart. In the adult heart, they are associated with a physiological turnover representing less than 1% of the total cardiomyocyte population per year (34). In physiological conditions in mammals, a switch between hyperplastic and hypertrophic growth occurs during the later stages of cardiac development (during the first postnatal week). This critical step ensures proper cardiac growth, but it is also hypothesized to be responsible for the loss of regenerative capacity in adults as cardiomyocytes lose their ability to proliferate. Hence, it is apparent that maintaining a pool of mononucleated diploid cardiomyocytes able to proliferate is an advantage to repair a damaged heart. In support of this notion, artificially inducing polyploidy in zebrafish cardiomyocytes reduces their ability to proliferate after injury. Indeed, *ect2* expression appears to be upregulated during regeneration, a protein known for its role in cytokinesis after DNA duplication (36). Furthermore, blocking Ect2 activity leads to cardiomyocyte polyploidisation which reduces their ability to proliferate and inhibits heart regeneration (36). Interestingly, maintaining a pool of diploid mononucleated cardiomyocytes using a mosaic trangenic line resulted in heart regeneration, suggesting that they are indeed the only form of cardiomyocytes able to proliferate and that a sufficient fraction of diploid mononucleated cardiomyocytes could be enough to fully regenerate the heart.

Apart from the relatively low physiological turnover, certain conditions are able to trigger cardiomyocyte cell cycle re-entry and proliferation, such as hypoxia, affecting the zebrafish heart following ventricle resection (16). This was later confirmed in mammals following the demonstration that the low-oxygenated environment present in zebrafish and mammalian fetuses/neonates was associated with less oxidative stress, a reduced DNA damage response and lower mitochondrial respiration, which is the preferred and most efficient metabolic pathway in aerobic conditions (when oxygen is more abundant). In comparison, late postnatal mammals present well-oxygenated heart tissue, more mitochondrial activity, reactive oxygen species (ROS) production and an elevated DNA damage response (37). Overall, this suggests that oxygen concentration conditions cardiomyocyte cell cycle arrest, highlighting a new regulatory mechanism of cardiomyocyte cell cycle reentry (38). Paradoxically, in adult mammals, MI induces hypoxia around the wound but is not associated with cell cycle reentry, suggesting it might not be the critical step to initiate a cardiomyocyte regenerative response.

Initial studies into the molecular regulation of heart regeneration have focused on the cell cycle and its associated factors. Among these factors, Mps1, a mitotic checkpoint kinase, was shown to be crucial for heart regeneration, providing the first evidence that cell cycle regulation was critical to induce regeneration after cardiac injury (7). Subsequently, *polo like kinase 1* (*plk1*), encoding another regulator of the cell cycle previously identified as being upregulated and particularly expressed within the wound border zone (39), was shown to be essential for zebrafish heart regeneration and was more specifically expressed in cardiomyocytes (9).

p38 MAP kinase (p38 MAPK) is also involved in cell cycle arrest in mammalian cardiomyocytes by repressing the expression of genes required for mitosis (40). p38 MAPK activity increases in the later stages of cardiac development, arresting cardiac growth and blocking cardiomyocyte proliferation. Subsequently, it was shown that p38 MAPK inhibition in adult mice can stimulate cardiomyocyte proliferation (40). In zebrafish, overactivation of p38 MAPK during cardiogenesis disrupts cardiac development, suggesting a similar role in cardiac growth (41). More interestingly, p38 MAPK is also expressed in adult zebrafish cardiomyocytes but is switched off (dephosphorylated) when cardiomyocytes proliferate (41). In constrat, overactivation of p38 MAPK completely blocks cardiac regeneration. Overall, it appears there are similarities regarding p38 MAPK activity and cell cycle arrest between zebrafish and mammals. However, unlike mammals after an injury, zebrafish cardiomyocytes appear to be able to turn off p38 MAPK activity in order to allow cardiomyocyte proliferation and ultimately, heart regeneration to occur.

#### Cardiomyocyte dedifferentiation

One of the the first steps in cardiomyocyte proliferation is dedifferentiation which is associated with a disassembly of the sarcomere which would otherwise impede cell division (9, 42, 43). *In vitro*, proliferation can be observed by maintaining isolated cardiomyocytes in culture for a few days. Using this approach, it appears that dedifferentiation is not only associated with partial sarcomere disassembly, but also with electrophysiological alterations. Indeed, the resting membrane potential of dedifferentiated cardiomyocytes appears to be much higher than in differentiated cells mostly caused by reduced K^+^ current (respectively −20/−10 mV after 4/7 days of culture *vs* -70 mV for freshly isolated rat cardiomyocytes) (44). This strong depolarization presumably affects the electrical activity of the cell and its ability to generate action potentials while on the other hand, promotes cardiomyocyte proliferation (45). In contrast, hyperpolarization appears to promote cardiomyocyte differentiation *in vitro* (46). *In vivo* cardiomyocyte dedifferentiation has also been widely described and is associated with an upregulation of fetal gene expression and dissasembly of the sarcomere and changes in cellular morphology (47). However, the intriguing question now is what triggers cardiomyocytes to redifferentiate *in vivo*? *In vitro,* once a cardiomyocyte has dedifferentiated it will not spontaneously redifferentiate which would indicate that extracellular factors/forces may be involved in this process during *in vivo* regeneneration.

#### Metabolic switch

**Figure 5:**
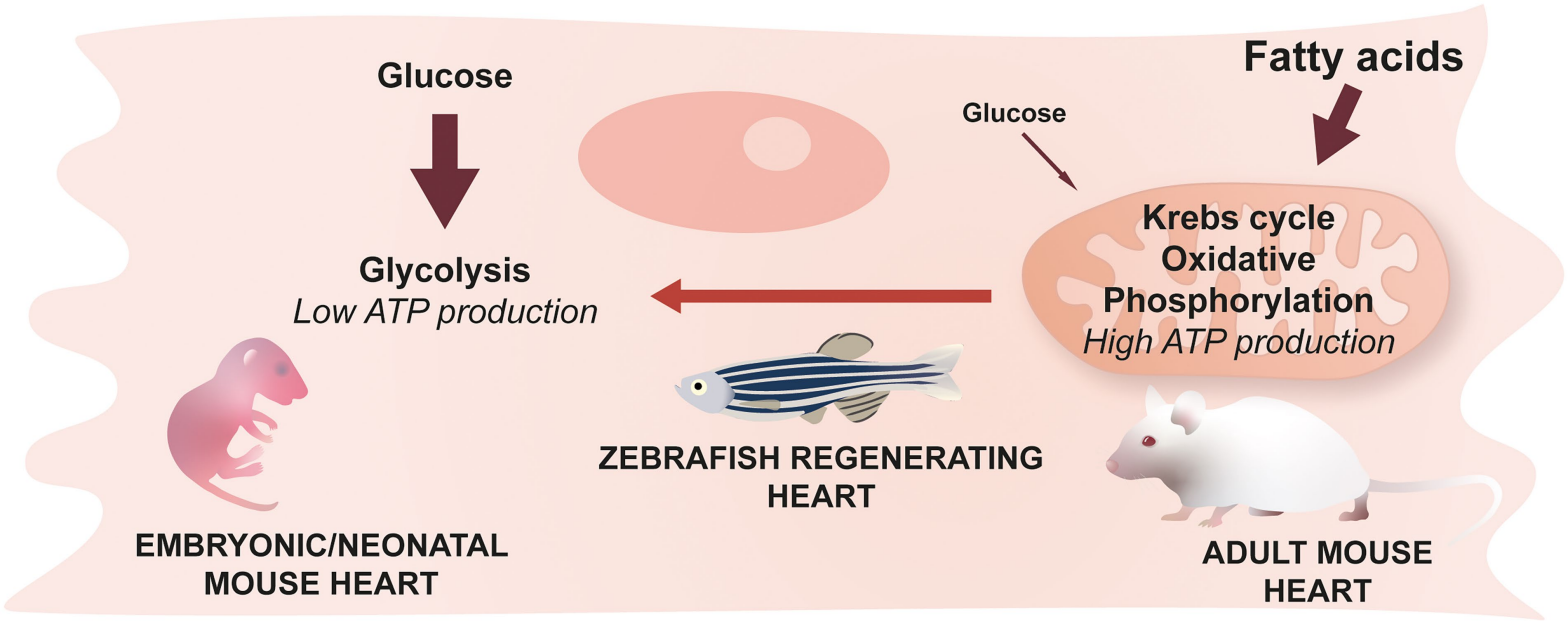
Cardiomyocyte metabolism & cardiac regeneration. Neonatal mice predominantly use glucose to generate energy while the main source of energy for adult mice comes from fatty acids and their oxidation. This switch occurring during the early stages of life is irreversible in mammals. Zebrafish cardiomyocytes, however, are able to revert back to a glucose-based metabolism during regeneration.

The heart is one of the most energy-consuming organs, requiring a large production of adenosine triphosphate (ATP) to function. Mitochondria are at the center of ATP production, but the pathways involved in ATP production differ depending on conditions such as environmental oxygen concentration. During adulthood in mammals, and under physiological conditions, large quantities of ATP are required and the most efficient pathway to produce it resides in fatty acid metabolism combined with aerobic mitochondrial activity. Interestingly, in mammals, a metabolic shift from glucose to fatty acid metabolism occurs in the first postnatal week, and coincides with increased mitochondrial activity, and the loss of regenerative potential (Figure 5) (37, 48). Overall, understanding this switch and identifying mechanisms to reverse it after an injury in adult mammals could provide therapeutic strategies to promote heart regeneration.

In addition to displaying signs of dedifferentiation characterized by less dense contractile apparatus (9), zebrafish cardiomyocytes located in the border zone after injury express transcription factors known for their role in embryonic heart development in mammals (18). In this respect, glucose metabolism also plays a critical role in the regenerative process as the inhibition of glycolysis (by a glucose analog) or pyruvate metabolism inhibits cardiomyocyte proliferation (18, 49). Among the genes involved in both embryogenesis and adult cardiomyocyte proliferation is the transcription factor Klf1. Inhibiting its activity impairs cardiac regeneration after injury and, in contrast, its overexpression leads to cardiomyocyte proliferation even in absence of injury. As a transcription factor, Klf1 stimulates the expression of genes involved in DNA replication and cell cycle progression while repressing the expression of proteins associated with mature cardiomyocytes (50). It is also involved in the metabolic switch by repressing genes involved in oxidative phosphorylation (50). Another factor involved in both metabolism and cell cycle reentry is Neuregulin 1 (Nrg1), a signaling glycoprotein which binds to ErbB2 and ErbB4 receptors shown to have mitogenic effects after injury (51) and to be involved in the switch from fatty acid to glucose-based metabolism occurring in zebrafish cardiomyocytes located at the border zone (52). Indeed, Nrg1/ErbB2 signaling mediates the expression of glycolysis genes in cardiomyocytes (18). Nrg1 is also produced by the endocardium and promotes dedifferentiation and proliferation in mammalian cardiomyocytes, however its expression is progressively reduced during the first week of postnatal life (53, 54), highlighting the similarities between mammalian cardiomyocyte development and zebrafish cardiomyocyte regeneration. NRG1 signaling is also involved in cardiomyocyte proliferation *in vitro*, as well as myocardial regeneration *in vivo* as demonstrated by improved cardiac function after MI in adult mice treated with NRG1 (55).

#### Cell communication

Cell–cell communication through signaling proteins, such as Nrg1, plays a crucial role in the cardiomyocyte regenerative response. Paired related homeobox1b (Prrx1b) is a transcription factor expressed in epicardium-derived cells (EPDCs) shown to be essential for zebrafish heart regeneration (56). Its expression increases in EPDCs around the wound and the epicardium boarding the injury during regeneration. In fibroblasts, it is responsible for the positive regulation of stress response and cell cycle-related genes while repressing the expression of ECM compounds and transforming growth factor β (TGFβ) signaling thus producing an anti-fibrotic effect (56). Interestingly KO of *prrx1b* in zebrafish leads to an inhibition of heart regeneration associated with a decrease of both *nrg1* expression and cardiomyocyte proliferation (56). Whether this is a direct or indirect effect remains to be investigated. Other signaling molecules which have been identified as necessary for cardiomyocyte proliferation after an injury include platelet-derived and insulin growth factors (Pdgf-b, Igf) (57), BMP (58) and Notch signaling (59).

#### Epigenetic elements

Recently, using ATACseq (assay for transposase accessible chromatin coupled to high throughput sequencing), it has been shown that chromatin accessibility varies in cardiomyocytes during heart regeneration in zebrafish (60). Among the enriched motifs uncovered, activator protein 1 (AP-1) motifs, binding sites recognized by AP1 transcription factors were the most accessible, suggesting this transcription factor complex could be involved in heart regeneration. In particular, AP-1 appears to regulate sarcomere disassembly in the border zone and also cardiomyocyte protrusion into the wound area (60). Another approach, ChIPseq (chromatin immunoprecipitation combined with DNA sequencing), can be used to determine whether a transcription factor is bound to chromatin under certain conditions. In this manner, it has been demonstrated that Histone H3 is bound to *lepb* enhancer region during zebrafish heart regeneration. Using a transgenic zebrafish reporter line it appears that this enhancer element is particularly active in the endocardium (61). As there is a growing interest for non-coding DNA regions, these techniques are particularly promising in the study of heart regeneration.

### It takes an entire troupe of actors to achieve regeneration

After over two decades of research into heart regeneration, it is apparent that cardiomyocytes are not the only cell type required for successful heart regeneration and that the cardiac interstitial cells also play a crucial role. A general timeline has also emerged, starting with a rapid immune reaction coupled with early angiogenesis into the injury site which allows the infiltration of other cells to clear the wound and prepare a matrice for cardiomyocyte proliferation.

#### The immune response

MI induces a ‘sterile’ inflammatory reaction as opposed to pathogenic inflammation. An initial pro-inflammatory response is triggered by the presence of debris and compounds released by dead cells. After the resolution of the inflammation, i.e., when the wound is cleared, a reparative phase starts. Acute inflammation is essential for the following reparative response in neonatal mice (62). Moreover, its duration is critical to limit the extent of damage caused by MI, for example reducing inflammation is associated with reduced pathological remodeling, in particular in the context of ischemia–reperfusion injury (63, 64).

Endogenous compounds called damage-associated molecular patterns (DAMPs) trigger inflammatory pathways after MI. These compounds, such as reactive oxygen species (ROS), can activate pattern recognition receptors (PRRs) present at the surface of immune cells, both circulating and resident, including macrophages, in order to trigger and propagate the immune response. These pathways are particularly important in matrix degradation as they are associated with the secretion of matrix metaloproteinases (MMPs) by necrotic and immune cells (neutrophils and macrophages). Overall, this contributes to the stiffening and remodeling of the ECM in adult mammals, switching from a fibrin-based to collagen-based matrix (65).

Currently, it appears that the origin of the macrophages involved in the MI-induced immune response differs between neonates and more mature mammals. In neonates, embryonic-derived resident macrophages proliferate to promote regeneration, in particular they promote a reduced inflammatory response and also secrete a variety of interleukins (ILs) such as IL10, IL6 and IL23 as well as pro-angiogenic factors (66, 67). Interestingly, IL6 appears to be associated with the inhibition of neutrophil recruitment in neonates but seems to have the opposite effect in adults (68). Conversly, in adults, macrophages are mostly monocyte-derived and have a proinflammatory profile which tends to promote fibrosis (66, 67). This difference of profile/origin alone seems to have a great impact on regeneration, for example, transfusing macrophages isolated from neonatal mice post apical resection into adult mice post MI leads to a significant increase in cardiomyocyte proliferation (69).

Macrophages are also required for regeneration in larval zebrafish following laser induced cardiac injury (19). Early after injury, macrophages display a proinflammatory profile, secreting proinflammatory cytokines. But, in contrast to mammals, their cytokine production reaches a peak a few hours after injury (as opposed to a few days). This proinflammatory phase is essential and its inhibition using anti-inflammatory glucocorticoid treatment leads to impaired regeneration and the presence of a fibrotic scar 30 dpa (70). But cytokine secretion is not the only macrophage activity at play during regeneration. Indeed, the production of MMPs to degrade the transient fibrotic deposition is essential and it has been shown that pharmacological inhibition of MMPs using GM6001 leads to fewer macrophages in the injured zone and drastically impairs regeneration (71). In particular it appears that *mmp14b*-expressing macrophages are recruited to the wound region during zebrafish heart regeneration and that the pharmacological inhibition of Mmp14 leads to disrupted macrophage infiltration within the wound and a failure to regenerate (72).

The role inflammation plays during heart regeneration also becomes apparent when comparing the response observed in regenerating zebrafish vs. another teleost, medaka, which cannot regenerate its heart. Transcriptomic analysis indicates discrepancies in the immune response with a strong and prolonged neutrophil response in medaka whereas zebrafish show an acute inflammatory response after injury that rapidly recedes. Overall, this suggests that the neutrophil/macrophage balance could play a critical role in the regulation of the immune response triggered by cardiac injury. Indeed, inhibiting the recruitment of macrophages in the zebrafish results in a failure to regenerate while conversely, stimulating macrophage recruitment in medaka is associated with fewer neutrophils present in the wound, as well as increased revascularization, cardiomyocyte proliferation and overall cardiac regeneration (73).

Neutrophils are recruited by the DAMPs and cytokines. They rapidly invade the wound after MI (74). Similar to medaka, a highly active neutrophil response, mostly pro-inflammatory and cytotoxic, is associated with pathological remodeling and a high mortality post MI both in humans and mice.

Monocytes are part of the leukocyte family and they adopt different profiles depending on the level of expression of Ly6C. Ly6C^hi^ monocytes (expressing high levels of Ly6C) are recruited by neutrophils very early during the pro-inflammatory phase and have phagocytic and proteolytic activity whereas Ly6C^low^ monocytes (expressing low levels of Ly6C) are involved in the resolution of the inflammation and secrete, among other things, vascular endothelial growth factor (VEGF) to promote angiogenesis in the injured area (75, 76).

Lymphocytes are part of the adaptative immune response and as such they can respond to autoantigens produced after an injury (77). CD8^+^ T cells have a cytolytic activity, whereas CD4^+^ T cells including CD4^+^ Tregs are involved in cytokine secretion and the regulation of the immune response (78). After MI, Tregs are known to reduce injury associated damage during pathological remodeling in mice (79). One explanation for the differences between neonates and adults regarding cardiac regeneration is based on the observation that in neonates, CD4^+^ T cells tend to generate Tregs in response to MI, thus promoting the resolution of the inflammatory phase, an ability they appear to lose around the second week of life (80).

It is apparent that the immune response associated with cardiac injury involves many factors and interactions with other proregenerative processes such as neovascularization of the wound. Although differences exist between the neonatal and adult immune responses, an inflammatory immune response is required for cardiac regeneration. Indeed, repressing the inflammatory phase after MI is not a solution *per se* to promote regeneration as inhibiting the immune response post MI in adults often leads to cardiac rupture, in both mice and humans (81, 82). Similarly, inflammation is essential to achieve regeneration in neonatal models and inhibiting this process impairs both cardiac function and regeneration (83). The contribution of immune cells is not limited to inflammation, they are essential for wound clearance and matrix remodeling, two essential processes involved in preserving cardiac integrity after MI. In mammalian models, achieving a faster resolution of inflammation seems to be a safer and more reliable solution to promote heart regeneration.

#### Endothelial cells

The endothelial cell response is essential to promote regeneration and in particular the fast revascularization of the injured zone.

In zebrafish, endothelial cell proliferation starts as early as 15 h after injury and vessels begin to form at 1 dpi (84). At this time, *vegfaa* expression increases, a growth factor required for revascularization of the wound region (84, 85). Disrupting Vegfaa activity results in the inhibition of cardiomyocyte proliferation, showing the importance of providing an optimal environment for cardiomyocyte proliferation and regeneration. Other factors such as Pdgfr-mediated signaling are also required for the endothelial regenerative response (86). Endothelial developmental transcription factors such as *tal1* also appear to be involved in triggering the revascularization required for cardiac regeneration, indicating that reverting back to a developmental state may not be just restricted to cardiomyocytes but may be a common feature of cardiac regeneration in general (72).

Lymphatic vasculature also plays an important role during cardiac regeneration by enhancing immune cell trafficking and in doing so, reducing inflammation (87). During the process of remodeling which occurs in the failing heart, angiotensin II (AngII) is expressed and associated with dysfunctions such as the misexpression of lymphatic vessel endothelial hyaluronan receptor 1 (LYVE1) and VEGFC. AngII injected animals display fewer lymphatic vessels with reduced diameters. However, overactivation of vascular endothelial growth factor receptor 3 (VEGFR3) is able to overcome the detrimental effects of AngII. Importantly, this is also associated with reduced fibrosis, a decrease in both hypertension and renal dysfunction known to be associated with AngII (88). In zebrafish, the disruption of Vegfc activity and its receptor Flt4, both of which are involved in lymphangiogenesis and the maintenance of the lymphatic vasculature, results in a failure to regenerate the heart after injury (65, 89). Although lymphatic vasculature increases after MI in mammals, lymphatic flow is reduced and does not function efficiently enough to reduce the damage of MI (90). However, it does represent a therapeutic target as an increased lymphatic flow can improve immune cell trafficking and wound clearing (90).

The endocardium also plays a dynamic role during cardiac regeneration, such as actively secreting pro-angiogenic factors like Notch and Raldh2 to support neovascularization (91–93). Notch signaling in particular appears to play a vital role during cardiac regeneration in zebrafish. After injury, Notch1a, Notch1b, and Notch2 receptors are strongly upregulated in endocardial and epicardial cells (59). Recently, it has been demonstrated that Notch signaling is not only essential for the endocardial response and wound revascularization but it also restricts inflammation and promotes cardiomyocyte proliferation (91, 92). Indeed, it is expressed around 3dpi and contributes to the suppression of the early inflammatory phase (91). At later stages of regeneration, Notch signaling stimulates cardiomyocyte proliferation by inhibiting Wnt signaling (*via* endocardial Wnt antagonist secretion) (92).

#### Fibroblasts, epicardium, and ECM

Fibroblast activation has been extensively characterized in the mammalian heart following injury and is associated with an elevated expression of *Acta2*, encoding α-smooth muscle actin. This is a characteristic of fibroblasts differentiating into myofibroblasts which are known for promoting fibrosis. Interestingly, zebrafish fibroblasts do not upregulate *Acta2* following cardiac injury (72). However, zebrafish activated fibroblasts and EPDCs do express high levels of *periostin b* (*postnb*), similar to the situation in mammals (72, 86). Indeed *Periostin* is also expressed after injury in neonatal mice and its inhibition blocks regeneration resulting in a fibrotic scar in the infarcted area (94).

Much like the immune response, it is apparent that although fibroblast activation is often associated with a pathology it is also essential for heart regeneration. Within the first 7 days after injury, fibroblasts are responsible for transient ECM deposition specific to regeneration, composed of fibronectin which fills the wound, as well as collagen (particularly after cryoinjury) in zebrafish (95). This is also the case in mammals, where the secretion of fibronectin and certain collagens by fibroblasts promote cardiomyocyte proliferation (96). Moreover, it is apparent that ECM stiffness impacts regeneration. Indeed, ECM stiffness increases with development and as such the fetal matrix is less rigid than the late neonatal ECM. Indeed, implantation of fetal ECM can improve cardiac function in regenerating P5 mice while adult ECM has the opposite effect and inhibits this process (97). This is also correlated with the presence of AGRIN, an ECM component present in the less rigid fetal matrix. AGRIN appears to be essential for heart regeneration in neonatal mice and its injection into adult mice after MI promotes cardiomyocyte proliferation (98). By interacting with dystroglycan complexes present at the surface of cardiomyocyte membrane, AGRIN triggers extracellular signal-regulated kinases (ERKs) phosphorylation and YAP (yes associated protein) translocation into the nucleus (98). YAP is a known regulator of heart growth, promoting cardiomyocyte hyperplasia and the expression of cell-cycle genes (99). Overall, it appears that ECM stiffness plays a fundamental role in regeneration by regulating cardiomyocyte proliferation, and, interestingly, the elasticity of the heart which rapidly decreases after birth, becoming 20 times more rigid during adulthood, which may also explain the loss of regenerative capacity (97). By communicating through ECM components, fibroblasts can promote cardiomyocyte proliferation, however, cardiomyocytes can also communicate with fibroblasts to stimulate heart regeneration. Indeed, *wntless* (*Wls*) expression in cardiomyocytes after an injury limits fibroblast proliferation as well as their activation and collagen production, representing another finely regulated process required for successful heart regeneration (100).

Epicardial cells also undergo a similar reversion in differentiation status as observed in cardiomyocytes and endothelium during regeneration. In particular, regenerating epicardium upregulates embryonic genetic programs such as *tbx18* and *raldh2* followed by cell proliferation between 3/7dpa (101, 102). This process is associated with epithelial-to-mesenchymal transition (EMT), resulting in the production of fibroblasts, as well as perivascular cells surrounding the newly formed vessels (101). Previous research in zebrafish and mice has proposed that the epicardium could in fact be a source of regenerated cardiomyocytes (103, 104). However, stringent lineage tracing indicates that this is not the case in regenerating zebrafish (9, 105). A recent lineage tracing study in salamanders does appear to show that this species may utilize the epicardium as a source of cardiomyocytes. Indeed, it appears that following injury aound 15% of the regenerated myocardium arises from epicardial cells (106). In comparison, the contribution of epicardium to zebrafish regenerated myocardium is negligible at best (9, 105). This indictaes that salamanders can regenerate substantial amounts of their hearts *via* a different mechanism to zebrafish and neonatal mice, however, this will need further verification.

### A recipe for success

Overall, it is apparent that cardiac regeneration is an intricate process involving the integration of a multicellular response. Inhibiting any part of this process results in a failure to regenerate the heart and demonstrates the complexity that is involved. However, it is also apparent from comparative studies that the mechanisms involved in zebrafish and neonatal heart regeneration are also present in adult mammals but are differentially regulated. For instance it has been demonstrated that adult mammalian cardiomyocytes can be induced to proliferate *in vitro* and that it is possible to trigger a regenerative, albeit reduced, response in adult mammals by activating or inhibiting some of the key factors previously identified in zebrafish and neonatal mice demonstrating the relevance of cardiac regeneration research and its potential impact in future therapeutic strategies (98, 107).

## Author contributions

LR and CJ conceived this review. LR wrote the manuscript. All authors contributed to manuscript revision, read, and approved the submitted version.

## Funding

This work was supported by INSERM, CNRS and the Laboratory of Excellence Ion Channel Science and Therapeutics (ANR-11-LABX-0015). Work in the Jopling Lab is supported by a grant from the “la Fondation Leducq” and from the ANR (contract ANR-20-CE14-003 MetabOx-Heart).

## Conflict of interest

The authors declare that the research was conducted in the absence of any commercial or financial relationships that could be construed as a potential conflict of interest.

## Publisher’s note

All claims expressed in this article are solely those of the authors and do not necessarily represent those of their affiliated organizations, or those of the publisher, the editors and the reviewers. Any product that may be evaluated in this article, or claim that may be made by its manufacturer, is not guaranteed or endorsed by the publisher.
